# Parameter estimation of neuron models using *in-vitro* and *in-vivo* electrophysiological data

**DOI:** 10.3389/fninf.2015.00010

**Published:** 2015-04-20

**Authors:** Eoin P. Lynch, Conor J. Houghton

**Affiliations:** ^1^School of Mathematics, Trinity College DublinDublin, Ireland; ^2^Department of Computer Science, University of BristolBristol, UK

**Keywords:** parameter estimation, spiking neurons, evolutionary algorithms, spike train metrics, auditory neurons

## Abstract

Spiking neuron models can accurately predict the response of neurons to somatically injected currents if the model parameters are carefully tuned. Predicting the response of *in-vivo* neurons responding to natural stimuli presents a far more challenging modeling problem. In this study, an algorithm is presented for parameter estimation of spiking neuron models. The algorithm is a hybrid evolutionary algorithm which uses a spike train metric as a fitness function. We apply this to parameter discovery in modeling two experimental data sets with spiking neurons; *in-vitro* current injection responses from a regular spiking pyramidal neuron are modeled using spiking neurons and *in-vivo* extracellular auditory data is modeled using a two stage model consisting of a stimulus filter and spiking neuron model.

## Introduction

It is difficult to find parameters for neuronal models that accurately replicate the responses seen in *in-vivo* extracellular data. One of the principle difficulties is that, for *in-vivo* data, the current input to the soma is generally unavailable. In this way, the model requires two components; one dealing with the current input to the soma and the other with the soma itself. The parameters describing these two components must be estimated from the spiking output and, in the case of sensory neurons, from the external stimulus. Since the underlying biological situation is complicated and non-linear there is always a trade-off which needs to be made between the predictive capability of the model and the ease with which the parameters can be estimated; this challenge is particularly acute when considering *in-vivo* data where the transformation from external stimulus to current input is non-linear and multi-dimensional.

Neuronal models vary in complexity from the integrate-and-fire model (Lapicque, 1907) through to fully biomorphically-realistic simulators based on the Hodgkin-Huxley model (Hodgkin and Huxley, 1952; Bower and Beeman, 1998; Hines and Carneval, 2002). In between these extremes are the two-variable spiking neuron models. These feature two dynamical variables; one models the membrane voltage while the other is typically a variable dealing with spike triggered adaptation. Dynamical systems analysis has shown that many of these simple models are capable of reproducing a wide range of the spiking behaviors commonly seen in real neurons (Izhikevich, 2003, 2006; Brette and Gerstner, 2005; Touboul and Brette, 2008) and model fitting studies have shown that they can accurately predict spike timings in *in-vitro* experiments where the input current to the soma is known (Brette and Gerstner, 2005; Clopath et al., 2007; Jolivet et al., 2008b; Kobayashi et al., 2009; Rossant et al., 2010). The crucial ingredient in these models, as far as modeling real data is concerned, is adaptation (Izhikevich, 2003; Kobayashi et al., 2009; Rossant et al., 2010). Commonly used examples of two variable neurons are the adaptive quadratic integrate-and-fire or Izhikevich model (Izhikevich, 2003), integrate-and-fire models with adaptation currents or adaptive thresholds (Geisler and Goldberg, 1966; Chacron et al., 2003), and the adaptive exponential integrate-and-fire (aEIF) model (Brette and Gerstner, 2005).

The various adaptive models have different strengths both in reproducing different types of spiking behavior and in ease of parameter estimation (Touboul, 2008; Izhikevich, 2010; Rossant et al., 2010). For example, the atIF neuron has only three parameters, making it relatively easy to fit; when modeling data from regular spiking cortical neurons, Rossant et al. (2010) found this model to be the most effective in comparison to other two variable spiking neuron models. On the other hand, the aEIF neuron, which presents a more difficult optimization problem with its eight parameters, has a wide dynamical range making it suitable to efficient simulations of networks with unusual neuron types (Naud et al., 2008; Touboul and Brette, 2008). Furthermore, its parameters have a more direct biophysical interpretation than those of many other simple models making it potentially useful in the histological characterization of cells. The Izhikevich model has particularly rich dynamics and with only four parameters and with a low computational cost it seems ideal for large scale neuronal network simulations (Izhikevich, 2006).

Several different approaches to fitting neuron models to electrophysiological data have been tested; (Van Geit et al., 2008), for example, provides a review of some recent progress in this area. As with optimization problems in general, the various methods employed on this problem can be broadly decomposed into two components: the objective function which is a similarity measure between the predicted and the experimental data and the particular optimization algorithm or method to be used to optimize the objective function.

The choice of optimization method is highly dependent on the model and the form of the experimental data, for example whether the data consists of spike trains or some other neuronal signal. If the input current to a neuron is not only known, but can be manipulated, model parameters can be estimated in an analytical fashion. For example, in Brette and Gerstner (2005), a variety of current pulses and ramps are used to isolate and identify the parameters in the aEIF model. On the other hand, if we restrict the choice of model to the leaky integrate and fire neuron with no adaptation then the parameters can be calculated by minimizing a convex function and gradient based methods are available: (Paninski, 2004; Paninski et al., 2004, 2005; Huys et al., 2006).

However, to develop a general purpose algorithm for neuron model optimization, a global heuristic optimization method is needed. This is a method which finds the global minimum, or maximum, of a function in a search space without using gradient information but instead using some carefully designed search heuristic. Examples of global heuristic search methods include stochastic search techniques such as simulated annealing and evolutionary algorithms.

In Clopath et al. (2007), the parameters for a two-compartment aEIF model were found using a simulated annealing approach to optimize the coincidence factor (Jolivet et al., 2008a), a discrete measure of spike train similarity which, roughly speaking, counts the number of spikes in one train that are coincident with a spike in another train to within some specified tolerance. Rossant et al. (2010) presented a general purpose model fitting method using a particle swarm algorithm, and using covariance matrix adaptation (Rossant et al., 2011), to optimize the coincidence factor and applied their algorithm to the INCF single neuron modeling competition data (Jolivet et al., 2008b).

Other fitness functions available for spike train data include the van Rossum distance (van Rossum, 2001), the Victor-Purpura metric (Victor and Purpura, 1996), the inter-spike-interval distance and SPIKE distance (Kreuz et al., 2007, 2011, 2013) and the correlation-based measure due to Schreiber et al. (2003). However, all of the available measures of spike train synchrony approach the problem differently and have been demonstrated to exhibit their own intrinsic bias and their own advantages (Kreuz et al., 2007; Naud et al., 2011; Houghton and Victor, 2012).

The van Rossum metric, which will be applied to neuron model fitting here, is a similarity measure which is computed by convolving spike trains with a filter to produce square integrable functions and then taking the standard *L*^2^ distance between these functions. The measure has several useful properties; it is continuous, which gives the algorithm sensitivity to small improvements in model accuracy; it does not require binning of spikes so it retains high fidelity to the temporal structure and it satisfies all the properties of a formal metric. With this in mind, it seems likely the van Rossum metric is as useful as many of the other available measures.

In this paper a general purpose evolutionary optimization routine is presented for calculating parameters for spiking neuron models responding to time varying signals which may be an input current waveform or an auditory stimulus. The optimization method is based on the genetic algorithm with real-value gene representations. The algorithm uses the van Rossum spike train metric as the fitness function.

Initially the algorithm is tested by fitting spiking neuron models to synthetic target data generated by other models with parameters known to lie in the search space. The algorithm is then applied to fitting models to experimental spike train data. Several two variable spiking neuron models are fit to *in-vitro* intracellular spike train recordings with known somatically injected currents. This data is taken from the Quantitative Single Neuron Modeling Competition (Jolivet et al., 2008b). A three variable model which is an extension of the aEIF neuron is presented and tested alongside the other models.

The algorithm is then extended to optimize an auditory neuron model, consisting of a cascade of a receptive field and a spiking neuron, by a tandem evolution approach. The model is fit to data consisting of song stimuli and extracellular spike train recordings from Zebra Finch. The full auditory model is compared to linear rate models in predicting the activity of the Zebra Finch auditory neurons responding to conspecific songs using two measures: the average coincidence factors and the van Rossum distances across trials.

## 1. Methods

### 1.1. Spiking neuron models

In this section we review the standard spiking neuron models and introduce a new spiking model which we found to be effective when applied to experimental data. Spiking neuron models, in contrast to more biophysically grounded models such as the Hodgkin-Huxley or Morris-Lecar neurons, are phenomenological models that reproduce neuron-like behavior with relatively few parameters. They exhibit neuron-like behavior in response to excitation but usually are not rooted in a careful balance of terms representing voltage gated ion channels acting on varying timescales, as in the Hodgkin-Huxley model. Rather, a sharp, discontinuous reset process at a spike threshold is used to model the down-sweep of the action potential.

This work investigates the fitting of models of this type with two variables. These models are expressed as either one or a pair of coupled ordinary differential equations with the general form as below. The first equation describes the time course of the membrane voltage *v* in response to an injected current *I* and adaptation variable *w*,

(1) $$\frac{dv}{dt}=f(v)-w+RI.$$

Here, *f*(*v*) is some function of *v* chosen to approximate the current response of a real neuron. *R* is a scaling factor for the current. The second equation describes the evolution of the adaptation variable,

(2) $$\frac{dw}{dt}=a(bv-w).$$

Since these models have no mechanisms to restore the voltage to resting after a spike, a sharp reset condition must be imposed at a cut-off voltage, *v*_*c*_, so when *v* > *v*_*c*_

(3) $$\begin{array}{l} v\to v_{r}\\ w\to w+\alpha \end{array}$$

The voltage is reset to a reset value, *v*_*r*_, and the adaptation variable is incremented by α.

In a real neuron, the membrane voltage rises sharply to a value greater than zero after a threshold is reached due to the opening of voltage-gated sodium ion channels; this effect is reproduced somewhat in exponential and quadratic integrate-and-fire models but not in the simple leaky integrate-and-fire model where there is no non-linearity around the voltage threshold. In other words, the exponential and quadratic models describe some part of the upswing of the spike but still require the manual insertion of part of the upswing and the down swing, the leaky integrate and fire model does not model any of the spike and do not account for the dynamics near threshold.

The various models in the literature are obtained by an appropriate choice of function *f*(*v*) in the differential equation for the voltage, Equation (1). For example, the basic IF model is defined by

(4) $$f(v)=-\frac{1}{\tau_{m}}(v-E_{L})$$

(5) $$\begin{array}{l} a=0 \end{array}$$

(6) $$\begin{array}{l} b-0 \end{array}$$

where τ_*m*_ is the membrane time constant. The aIF model is defined by

(7) $$f(v)=-\frac{1}{\tau_{m}}(v-E_{L})$$

(8) $$a=\frac{1}{\tau_{w}}$$

(9) $$\begin{array}{l} b=0 \end{array}$$

where τ_*w*_ is the adaptation time constant.

A separate but related type of model, involving an adaptation of the spiking cutoff voltage replaces the second equation in the previous pair, Equation (2), with a similar equation for *v*_*c*_,

(10) $$\tau_{t}\frac{dv_{c}}{dt}=bv-v_{c}$$

and instead incrementing *v*_*c*_ during the reset process by α.

The aEIF model is defined by

(11) $$f(v)=\frac{1}{\tau_{m}}\left[(E_{L}-v)+\Delta_{T}\exp^{\left(v-v_{T}/\Delta_{T})\right)}\right]$$

(12) $$a=\frac{1}{\tau_{w}}$$

*E*_*L*_ is the effective resting potential of the neuron and Δ_*T*_ is a slope factor for the exponential term. *v*_*T*_ is the threshold voltage. When *v* passes this threshold its value increases exponentially and spike initiation is almost unavoidable. This mimics the effect of the opening of voltage gated sodium ion channels in real neurons (Brette and Gerstner, 2005).

The adaptive quadratic integrate-and-fire (aQIF) or Izhikevich model (Izhikevich, 2003) is defined by

(13) $$f(v)=0.04v^{2}+5v+140$$

and a reset condition as before although Izhikevich refers to the reset voltage as *c* rather than *v*_*R*_ and the increment in the adaptation variable as *d* rather than α. Spike cut-off occurs at a fixed value of 30 mV. The cutoff value has been shown to be critical to the spiking behavior of this model (Touboul, 2009).

Possible extensions of these two variable models are inclusions of multiple time scales of adaptation in either the threshold or the adaptation current by the inclusion of additional equations of the form of Equations (2) or (10). Promising results have been achieved with an approach like this. Kobayashi et al. (2009), for example, introduced a leaky integrate-and-fire model with multiple time scales of adaptation in the spike threshold, the multiple adaptive timescales (MAT) model. Their model consists of a non-resetting leaky integrate-and-fire neuron in which the spike threshold is increased each time the spike threshold is reached.

Overall, it seems that adaptation is both crucial to model performance and occurs on multiple time scales. In Kobayashi et al. (2009) the predictive powers of a Hodgkin-Huxley model, leaky integrate-and-fire model and a Spike Response model with an adaptive threshold were compared to the MAT model using *in-vitro* conductance based test data. The coincidence factor, Γ, scaled by the intrinsic reliability, Γ_*i*_ is used to calculate an effective predictive score, Γ_*A*_ = Γ/Γ_*i*_. The Hodgkin-Huxley model achieved a predictive score of Γ_*A*_ = 0.51 ± 0.26. The leaky integrate-and-fire model and spike response model with a single scale adaptive thresholds yielded Γ_*A*_ = 0.66 ± 0.26, and Γ_*A*_ = 0.70 ± 0.26, respectively. The multiple adaptive timescales model with three scales of adaptation achieved the best predictive score of Γ_*A*_ = 0.89 ± 0.21. In otherwords, the effectiveness of the model increases as the amount of adaptation is increased and the inclusion of adaptation is more useful at improving accuracy than the biophysical replication of short timescale channel dynamics of the Hodgkin-Huxley equation.

Here we propose an extension of the aEIF model which includes a second level of adaptation in the spike threshold parameter. The model has similar sub-threshold dynamics to the original aEIF model, however the extra dynamical equation allows for the threshold to increase after spiking. We call this multiply adaptive exponential integrate-and-fire model the a^2^EIF model. The dynamical equations of the model are

(14) $$\tau_{m}\frac{dv}{dt}=E_{L}-v+\Delta_{T}\exp^{\left(v-v_{t}/\Delta_{T}\right)}-w+R\tilde{I}$$

(15) $$\tau_{w}\frac{dw}{dt}=bv-w$$

(16) $$\tau_{t}\frac{dv_{t}}{dt}=V_{t0}-v_{t}$$

The parameters τ_*t*_ and β are the time constant of threshold adaptation and spike-threshold increment respectively. The reset occurs when the voltage reaches *v*_*c*_ and has an effect on each of the three dynamic variables;

(17) $$v>v_{c}\{\begin{array}{l} \begin{array}{l} v\to v_{r}\\ w\to w+\alpha \\ v_{t}\to v_{t}+\beta \end{array} \end{array}.$$

where *c* is a constant set at the initial threshold voltage, typically ≈ −50 mV. The model differs from the MAT model and other adaptive threshold models in that the adaptation occurs in the threshold voltage parameter of the exponential term rather than the spike time recording threshold voltage. The *v*_*t*_ parameter defines the membrane voltage at which the exponential term becomes positive and initiation of a spike is almost certain.

### 1.2. Auditory model

In this section the other part of modeling the *in-vivo* situation is discussed, the transformation of a natural stimulus to a time varying signal which may be a firing rate or an input current. We demonstrate how to estimate this signal in the case of an auditory stimulus and use it as part of a spiking model of an auditory neuron.

The responses of auditory neurons to auditory stimuli are commonly characterized using spectro-temporal receptive field (STRF) models (Aertsen and Johannesma, 1981; Theunissen et al., 2000; Sen et al., 2001; Olshausen and Field, 2004; Woolley et al., 2005; Gill et al., 2006). The STRF is a kernel, *h*(τ, ω), which describes how an auditory neuron responds to stimuli by taking a weighted sum over different latencies and frequencies. Convolving the STRF with the stimulus *s*(*t*, ω) gives an estimate $\tilde{r}(t)$ of the neurons firing rate:

(18) $$\tilde{r}(t)=\int_{\Omega }\int_{0}^{T}h(\tau ,\omega )s(t-\tau ,\omega )d\tau d\omega .$$

In the linear case, the receptive field is estimated by formulating a least squares problem between the estimated firing rate, $\tilde{r}(t)$ and the actual experimental firing rate, *r*(*t*). *r*(*t*) is typically approximated by the peri-stimulus time histogram which is the ensemble averaged rate across multiple trials of the same stimulus and this is the method applied to the experimental data sets used here. The problem is to minimize 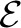,

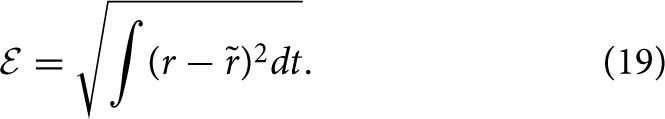



Several methods have been applied to the solution of Equation (20), with normalized reverse correlation being commonly used (Theunissen et al., 2001; Ringach and Shapley, 2004; David et al., 2007). Gradient descent, coordinate descent (Theunissen et al., 2001) and boosting (Friedman et al., 2000; David et al., 2007; Willmore et al., 2010) are also popular choices. More recently a generalized linear model approach was proposed by Calabrese et al. (2011).

The STRF calculation used here is based on the pseudo-inverse technique commonly used elsewhere (Theunissen et al., 2001; David et al., 2007). The method proceeds as follows: the linear model, Equation (18), can be written in a discrete form as

(20) $$\tilde{r}(t)=\sum_{i=0}^{N-1}\sum_{k=0}^{M-1}h_{t}(i,k)s(t-i,k)$$

where *i* is an index over *N* points in time and *k* is an index over *M* frequency values. The indexes of Equation (20) can be vectorized so that only one sum over the spatial and spectral dimension of the stimulus is required.

(21) $$\tilde{r}(t)=\sum_{i=1}^{N\times M}h(i)s_{t}(i)$$

where *h* = [*h*_1_, …, *h*_*M* × *N*_]^*T*^ is the vector of STRF coefficients. Minimizing $\langle {\left(r-\tilde{r}\right)}^{2}\rangle$ gives the solution for the receptive field vector *h*,

(22) $$h={\left(S^{T}S\right)}^{-1}C_{sr},$$

where (*S*^*T*^*S*) is the (*NM* × *NM*) stimulus auto-correlation matrix and *C*_*sr*_ is the cross correlation vector between the stimulus and the response. The inversion of the stimulus auto-correlation matrix requires careful consideration.

The singular value decomposition of the auto-correlation matrix (*S*^*T*^*S*) will typically show that it has many very small or zero eigenvalues corresponding to the low variance stimulus dimensions seen in natural stimuli. Inversion of the matrix will amplify the noise from these stimulus dimensions in the STRF. Thus, (*S*^*T*^*S*)^−1^ is computed by first taking the singular value decomposition of *S*^*T*^*S* and constructing a pseudo-inverse for it using a regularization. The singular value decomposition of *S*^*T*^*S* can be written

(23) $$S^{T}S=U\Sigma V^{*}$$

where Σ is a diagonal matrix of the singular values and *U* and *V* are unitary square matrices. The pseudo-inverse of *S*^*T*^*S* is then given by

(24) $${\left(S^{T}S\right)}^{+}=V\Sigma^{+}U^{*}.$$

A regularization strategy is applied to the singular value matrix, Σ, during inversion to form the regularized pseudo-inverse. It requires selection of a single hyper-parameter λ which specifies the tolerance of the regularization and is optimized using a cross-validation procedure. The number of stimulus dimensions to preserve, *m*, is computed by

(25) $$m=\text{arg max}\frac{\sigma_{1}+\sigma_{2}+\ldots +\sigma_{m}}{\sigma_{1}+\sigma_{2}+\ldots +\sigma_{MN}}<\lambda$$

If σ_*i*_ are the singular values in Σ, then after this regularization procedure the non-zero elements of the inverse of Σ, that is Σ^+^, are given by

(26) $$\Sigma^{+}=\text{diag}\left(\frac{1}{\sigma_{1}},\frac{1}{\sigma_{2}},\ldots ,\frac{1}{\sigma_{m}},0,\ldots ,0\right)$$

Receptive field models estimate firing rates and not spike trains. However, the firing rate can be used to generate spike trains using some spike generation mechanism. For example, an inhomogeneous Poisson process can be used to generate spike trains in a probabilistic manner from the rate. Here, a two stage model is used in which a receptive field model is adapted to produce an estimate of the input signal to a two-dimensional spiking neuron model. This is a deterministic model of spike generation for auditory neurons *in-vivo*.

(27) $$\frac{dv}{dt}=f(v)-w+\tilde{I}$$

(28) $$\frac{dw}{dt}=a(bv-w)$$

(29) $$\tilde{I}(t)=C\int_{\Omega }\int_{0}^{T}h(\tau ,\omega )s(t-\tau ,\omega )d\tau \;d\omega .$$

$\tilde{I}$ is an estimate of the input current signal and *C* is some constant.

**Figure 1:**
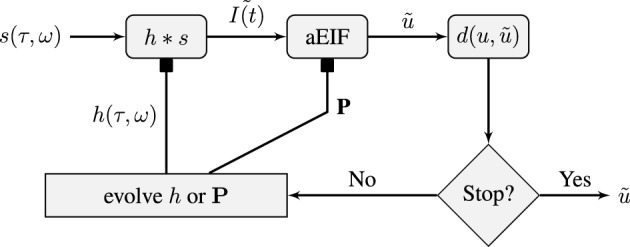
**Schematic of the flow of the STRF-Neuron cascade model optimization algorithm.** The input stimulus, *s*(τ, ω), is a spectro-temporal representation of a sound. It is convolved with a STRF, *h*, which forms the input to an aEIF neuron. The predicted spike train, $\tilde{u}$, is then compared against the training data and validation data spike trains using the van Rossum distance, *d*(*u*, $\tilde{u}$). The population results on the training data are then used to evolve either the population of STRFs or the population of aEIF parameter sets, **P**, independently. This is done in tandem and in real simulations, a regime of alternating between 50 iterations of evolving each population was used. The result against the validation data is used for tracking convergence and for convergence criteria; the algorithm can be stopped after a maximum number of specified iterations or when *d*(*u*, $\tilde{u}$) < ϵ then we have a sufficiently accurate solution.

We shall attempt to fit this model to *in-vivo* extracellular spike train data using an evolutionary algorithm which is described in the next section. Our approach will be to use a tandem evolution method to alternately evolve the set of neuron parameters and the the set of STRF parameters for some number of iterations. This is illustrated in Figure 1 which shows a flowchart of the model and the optimization process. The three variable a^2^EIF model will not be used in the two stage auditory model because it presents a more challenging optimization problem, as will be shown later, and its parameters have not been well studied. Instead, the standard aEIF neuron will be used.

### 1.3. Optimization algorithm

In this paper a genetic algorithm is used; it is customized to parameter discovery for the two stage spiking model. The genetic algorithm is a heuristic optimization method inspired by Darwinian evolution, first introduced in Holland (1973). The method starts with an initial population of parameter sets which represent potential solutions to the problem. The parameter sets in the population are ranked based on the fitness measure to be optimized, for example, how well a model predicts an experimental spike train. Selection and breeding are used to produce a new generation of parameter sets with most of the good characteristics of the previous generation and, typically, a higher average fitness. The process is continued until a generation produces a set of parameters that sufficiently optimize the problem or until a time limit is reached.

More precisely, the genetic algorithm is used to find a parameter set **x** ∈ **R**^*n*^ that minimizes a fitness function *f* (**x**) ∈ **R**; **x** may be constrained to lie in some feasible region **S** ⊂ **R**^*n*^. It is an iterative, population-based method in which a population set *P*_0_ = {**x**_1_, …, **x**_*N*_} of candidate parameter sets, **x**_*i*_ ∈ **S**, is randomly initialized and successive generations of parameter sets *P*_1_, …, *P*_*i*_ are iteratively generated from the previous generation using a number of pre-defined rules to combine and randomly modify parameter sets and form new parameter sets.

The fitnesses of the parameter sets in each generation are calculated and ordered in ascending order. To create a new generation set, *P*_1_, first, the fittest *k* members of *P*_0_ are selected and copied exactly to form *k* elements of *P*_1_. This procedure is referred to as *elitism* and *k* is a run time specified parameter. The purpose of the elitism step is to ensure that good solutions, once found, are not lost.

Next, breeding is used to replace the other *n* − *k* members. The first step in breeding is selection. There are a number of commonly used methods for this; here the roulette wheel selection method is applied which essentially ensures that parents are selected from the population randomly but with probabilities proportional to their fitness values. Once crossover is complete, a small percentage of the members of *P*_1_ are randomly selected with some mutation probability, typically ≈5%, to undergo mutation.

The algorithm developed here uses a non-uniform mutation operator inspired by those commonly presented in, for example, Michalewicz (1996). If a neuron model parameter set is selected for mutation, a single parameter is chosen and rescaled by 1 + *r*, where *r* is a Gaussian random number of mean zero and variance ϵ_*m*_. The value of ϵ_*m*_ decreases with successive iterations. This causes the algorithm to search the parameter space widely in early iterations and to more finely tune its search in later generations. 0.2 was determined to be a roughly optimal initial value for ϵ_*m*_ through trial and error as, on average, the best convergence was observed with this value.

The differential equations governing spiking neuron models are invariant under a number of scaling transformations. Indeed many of the models can be rescaled and their number of parameters reduced. For example, Touboul and Brette (2008) showed that rescaling of an aEIF neuron reduced it to from eight to four parameters. The genetic algorithm traverses this invariance and any set of parameters found by the genetic algorithm can be rescaled to biologically plausible ranges. In the model fitting studies here, we have ignored these invariances; instead, the algorithm uses user specified ranges for each parameter to draw initial values for the population.

There is also an ambiguity in the overall scale of $\tilde{r}(t)$, the output of the STRF model, when $\tilde{r}(t)$ is regarded as a rate the overall scale can be fixed to give the correct average firing rate. Here $\tilde{r}(t)$ is used as the input to the spiking neuron model and the problem of finding the over scale is separated from the problem of determining the shape of the STRF by constraining the STRF to have an *L*^1^ norm equal to one. The correct scaling factor is then regarded as one of the parameters describing the neuron model and is optimized along with the other neuronal parameters.

The parameters of each model that was presented in Section 2.1 and 2.2. are summarized in Table 1. These will be optimized by the genetic algorithm. The table lists each models' parameter set, number of parameters and the storage format used as well as the total size in memory of a chromosome.

**Table 1:** **Parameters and gene size for each model**.

**Model**	**Optimized parameters**	**Fixed parameters**	***N***	**Type**	**Gene size**
atIF	τ_*m*_, τ_*t*_, α, *b*, *R*	*E*_*L*_, *V*_*c*0_	5	float	20 bytes
aIF	τ_*m*_, τ_*w*_, α, *R*	*E*_*L*_, *V*_*c*_	4	float	16 bytes
aEIF	τ_*m*_, τ_*w*_, *E*_*L*_, Δ_*T*_, *V*_*T*_, *b*, α, *V*_*r*_, *R*	*V*_*c*_	9	float	36 bytes
*a*^2^EIF	τ_*m*_, τ_*w*_, τ_*t*_, *E*_*L*_ Δ_*T*_, *b*, α, β, *V*_*r*_, *V*_t0_, *R*	*V*_*c*_	11	float	44 bytes
aQIF	*a*, *b*, *c*, *d*	*V*_*c*_	4	float	16 bytes
STRF	20× 40 matrix *h*(τ, ω)		800	float	3200 bytes

Table showing the number of parameters of each model and the parameters being optimized for each model together with data type and gene size. Single precision floating point representations were generally used because the Nvidia Tesla C1060 GPU used to run the simulations was from an early generation of Nvidia GPUs which did not have full support for double precision. Switching to double precision would be relatively straightforward if required.

### 1.4. Fitness function

Here the van Rossum metric (van Rossum, 2001) is used as the fitness function. The van Rossum metric is a continuous measure of the dissimilarity between two spike trains. The metric is computed by convolving spike trains with a filter to map the trains to square integrable functions. A spike train

(30) $$u=\{u_{1},u_{2},\cdots ,u_{m}\}$$

is mapped to a real function, *f*(*t*; **u**) using a filter *h*(*t*):

(31) $$u\mapsto f(t;u)=\sum_{i=1}^{m}h(t-u_{i}).$$

Here, an exponential kernel is used:

(32) $$h(t)=\{\begin{array}{ll} 0 & t<0\\ \frac{2}{\tau }e^{-t/\tau } & t\geq 0 \end{array}.$$

where τ is a timescale which determines the relative sensitivity of the metric to fine temporal features in the spike trains. The van Rossum distance between two spike trains **u** and **v** is then the *L*^2^ metric between their respective functions, that is

(33) $$d(u,v)=\sqrt{\int dt{\left[f(t;u)-f(t;v)\right]}^{2}}$$

An efficient algorithm for computing the metric, presented in Houghton and Kreuz (2012), was employed in our simulations.

**Figure 2:**
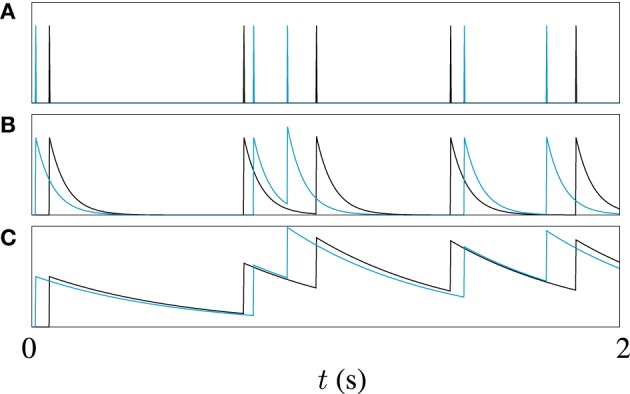
**An illustration of the effect of filter width on van Rossum metric; (A) Shows two spike trains, one in black, the other in blue, (B) Shows the functions obtained by filtering the spike trains with a causal exponential with a time constant of τ = 10 ms, and (C) Shows the same trains filtered with time constant of 50 ms, 5 × τ**.

Often, some sort of metric clustering based optimization routine is used to pick the best timescale τ for a data set (Victor and Purpura, 1997). Here, however, different timescales are used to explore different points in the spike trains in our genetic algorithm. Different values of τ make the metric sensitive to features on different timescales in the spike trains. A long timescale allows similarities in large scale features to be detected, for example, the mean firing rate, while a short timescale will be more sensitive to the differences between very similar spike trains. This is illustrated by the example in Figure 2. Figure 2A shows two spike trains. In Figure 2B the spike trains have been filtered to form functions with a short timescale. The functions formed by filtering the spike trains only significantly overlap when spikes from each train are close to each other. Figure 2C demonstrates that for a long timescale, very different spike trains can produce relatively similar functions. To demonstrate the effect of this on model optimization, we will later present the result of using a variable timescale which starts at a value of the order of the length of the target data set and is then reduced to a value roughly equal to the mean inter-spike interval.

It is convenient to introduce objects that play the role of “average” spike trains. Following Julienne and Houghton (2013) the average function is the mean of the functions obtained by filtering each train in a set and the central spike train is a spike train constructed so that its filtered function is close to the average function. These objects provide an efficient alternative to fitting to many trials of the same stimulus and a means of noise reduction similar to peri-stimulus time histogram estimates of the firing rate but with the result of exact spike times.

In addition to the van Rossum metric, we use the the coincidence factor as benchmark test for quantifying the performance of neuron models since it is widely used for this purpose (Jolivet et al., 2008a). Roughly speaking the coincidence factor counts the fraction of spikes in one spike train that are coincident to a spike in a target spike train to within some temporal tolerance, δ. The coincidence factor is defined by

(34) $$\Gamma =\frac{2}{1-2\delta f}\left(\frac{N_{c}-2\delta N_{e}f}{N_{e}+N_{m}}\right)$$

where *N*_*c*_ is the number of coincident pairs between the two spike trains, *N*_*e*_ and *N*_*m*_ are the number of spikes in the experimental and model spike trains respectively. δ is the coincidence window which defines a coincidence; if the absolute time difference between two spikes is less than δ then they are coincident. *f* is the average firing rate in the experimental target. The normalization factor ensures that Γ ≤ 1 and Γ = 1 corresponds to a perfect match at the given δ value. The intrinsic reliability of the target data set, denoted Γ_*i*_, is the average inter-trial coincidence factor. It is a measure of the underlying unreliability of the spike trains and can be used to rescale model coincidence factors into an effective performance factor. If a model achieves a performance factor of Γ/Γ_*i*_ ≥ 1 in predictions on a validation set then it can safely be concluded that the model is making predictions to within the variability of experimental data set.

### 1.5. Numerical experiments

A number of numerical experiments were designed and performed to test the effectiveness of our algorithm at parameter estimation and neural response prediction in different situations. The details of these experiments are described in this section.

To test the effectiveness of the algorithm in isolation, synthetic target data generated by a spiking neuron model target was used. Here it was expected that a model with a high coincidence factor with the target data could be found by the algorithm since it was known in advance that a model with the same dynamics exists.

Twenty experimental runs were performed as follows. Using the aEIF model, a sample parameter set was used to simulate 4 s of spike train data in response to a random input current signal. This synthetic data set, consisting of the spike train and current signal, was then used as the target for the genetic algorithm. For this purpose, the data set was divided in two with the first 2 s being for training and the final 2 s for validation. The genetic algorithm was initialized with a population of 240 neuron models each with a parameter set drawn randomly from its feasible region. The target parameters are indicated in Table 2. The feasible regions of each are quoted in the Supplementary Material accompanying this article.

**Table 2:** **Target parameters and found parameters**.

	**τ_*m*_**	**τ_*w*_**	***b***	***V*_*T*_**	***V*_*R*_ = *E*_*L*_**	**α**	**Δ_*T*_**
Range	[3, 17] ms	[36, 204] ms	[0.0003, 0.0017]	[−70, −20] mV	[−120, −50] mV	[0.3, 1.7] mV	[0.5, 3] mV
Target	10 ms	144 ms	0.001	−50 mV	−70 mV	1 mV	2 mV
Discovered	11 ± 2.6 ms	145.5 ± 4 ms	0.0011 ± 0.0004	−50 ± 7 mV	−70 ± 1 mV	1± 0.1 mV	1.75 ± 0.4 mV

The table shows the values of parameters of the adaptive exponential integrate-and-fire model which were used to generate synthetic target data in the model finding runs together with the search range used by the genetic algorithm for each parameter and finally the average parameter values discovered by the algorithm plus or minus a standard deviation across 20 runs. The average coincidence factor between the discovered models and the target data was 0.98±0.05 at a coincidence window width of Δ = 0.5 ms.

We chose a population size of 240 as it is the number of cores on the NVidia CUDA GPU we ran the code on. The algorithm was set to run for 1000 iterations to allow full convergence and the evolution of the population best van Rossum distance and coincidence factor when compared against the validation data set was observed.

It was also investigated here whether using a variable timescale in the van Rossum distance fitness function results in a more effective optimization search than a fixed timescale. Three test case optimization runs were performed; in the first the algorithm was set to use a timescale which starts at a value half the length of the target data set and is reduced with subsequent iterations to a value equal to the mean inter-spike interval. The decrement in τ is logarithmic with the base being calculated using the maximum number of genetic algorithm iterations, *N*. In the second and third test cases, the van Rossum distance was set to have fixed timescales equal to half the data set width and the mean inter-spike interval respectively. For each test case, the algorithm was run 20 times for 200 iterations and the convergence behavior was observed.

#### 1.5.1. Fitting to *In-Vitro* intracellular data

**Figure 3:**
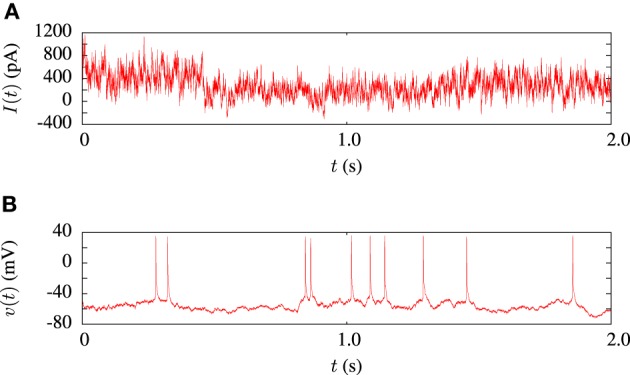
**Sample of data from the *in-vitro* L5 pyrmadial neuron experiment. (A)** Shows 2 s of current input which was injected during the experiments. **(B)** Is the corresponding voltage trace from one of the 13 experimental trials.

The model fitting procedure was tested on the publicly available data from challenge A of the INCF quantitative single neuron modeling competition, 2009 (Gerstner and Naud, 2009). The data set consisted of a 38s voltage recording from regular L5 pyramidal neuron of a rat responding to an *in-vivo* like current injection. Random current injection starts at 17.5 s into each trial and continues for the remainder. A 2 s sample of the input current and a corresponding voltage trace is illustrated in Figure 3. In order to compare the performance of our algorithm in *in-vitro* data modeling against a similar study, we tested out algorithm using the same data and models as Rossant et al. (2010).

The 20.5 s segment of current injection data set was divided up as follows; the first 10.5 s was used for model fitting and the the last 10 s for validation. The spike times were extracted from the voltage trace by using interpolation to find the times in the trace at which the voltage crossed a threshold value.

The algorithm was run for each of five neuron models to find the best parameters in each of the aIF, atIF, aEIF, a^2^EIF, and Izhikevich models. The algorithm was set to run for 800 iterations on each trial. The best average inter-trial coincidence factor obtained from each run and the corresponding parameters were recorded. We also studied the MAT model using the parameters described in Kobayashi et al. (2009).

#### 1.5.2. Fitting to *In-Vivo* extracellular data

**Figure 4:**
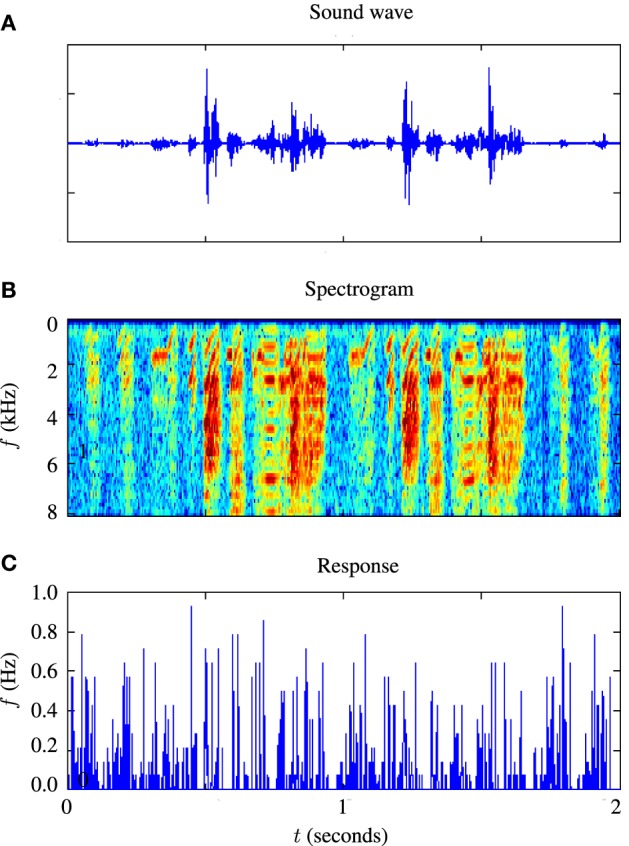
**Illustration of stimulus and response from an example *in-vivo* cell. (A)** Shows the raw sound waveform of 2 s of Zebra Finch song, **(B)** shows a spectrographic representation of the sound, the y axis indicating the frequency in kHz, and **(C)** shows the peri-stimulus time histogram of the response of the cell.

For testing the auditory model, we used the publicly available Zebra Finch data set, “aa-2,” available on the collaborative research in computational neuroscience (CRCNS) website (Gill et al., 2006; Amin et al., 2010; Theunissen et al., 2011). The data set consists of 445 sets of extracellular recordings of live anesthetized Zebra Finch responding to both conspecific songs and noise. Each set consisted of between 10 and 20 auditory stimuli with an average duration of $\tilde{2}$ s and corresponding sets of spike train responses to 10 repetitions of each stimulus. The experiments which produced these data are described in Gill et al. (2006). An example of 2 s of input and response data is illustrated in Figure 4. Figure 4A is a sound waveform, Figure 4B shows the sound in spectrographic form and Figure 4C shows the corresponding peri-stimulus time histogram obtained from extracellular recordings of the response to 20 repetitions of the sound stimulus.

Data consisting of 110 of the 445 electrode data sets was selected from the full set for testing of the full model optimization routine as follows; an ensemble average intrinsic reliability, 〈Γ_*i*_〉, was first estimated for each of the 445 cells in the data set by computing the intrinsic reliability between trials for each stimulus with δ = 2 ms and then averaging across stimuli. The cells with 〈Γ_*i*_〉 greater than 0.1 were then selected for testing. A coincidence factor of 0 would correspond to random chance so 0.1 was deemed a sensible limit to eliminate highly noisy data.

The spectro-temporal receptive field of each cell in the cohort was computed using the normalized reverse correlation method presented earlier using the STRFlab Matlab toolbox (David et al., 2010). The STRFs were chosen to have 20 frequency bands between 0 and 8 kHz and 40 evenly spaced time points with a total temporal width of 40 ms. The STRF for each cell was computed using the Lyon's cochleagram representation of the stimulus (Lyon, 1982). Gill et al. (2006) demonstrated that this representation yields STRFs with the highest predictive accuracy on this data set when compared against STRFs computed from log spectrogram and wavelet representations. The Lyon's cochleagrams were computed using the Auditory Toolbox written by Malcolm Slaney (Slaney, 1998). These STRFs were used as initial conditions in the genetic algorithm.

Finally, the full model optimization method was applied to the 110 data sets from the experimental data set as follows. First, each data set was split in half to form training and validation sets. For example, in the sets with 20 songs, 10 were used for validation and 10 for training. The algorithm was run for a maximum of 600 iterations on each cell, using the average van Rossum function obtained by summing all the spike trains as the target. It was observed that the algorithm generally stopped converging after a few 100 iterations. This is how a limit of 600 iteration was decided upon; no significant improvement was likely beyond this.

The performance of the full model spike predictions were benchmarked against the STRF rate models realized as a set of spike trains by generating spikes from the rate using an inhomogeneous Poisson process (Lewis and Shedler, 1979). Both the van Rossum distance and the coincidence factor were used as performance measures. The expected coincidence factors were evaluated between an inhomogeneous Poisson process with rate *r*(*t*) and the set of experimental trials as follows. Following on from Equation (34) this is

(35) $$\langle \Gamma \rangle =\frac{\langle N_{c}\rangle -2f\delta N_{e}}{\frac{1}{2}\left(N_{e}+\langle N_{m}\rangle \right)}\frac{1}{1-2f\delta }$$

The expected number of spikes generated by the inhomogeneous Poisson process, 〈*N*_*m*_〉, is just the integral of the rate over the time $\int_{0}^{T}\tilde{r}(t)dt$. The value of 〈*N*_*coinc*_〉 was estimated using a Monte Carlo like method in which the number of coincidences was evaluated for 100 simulated Poisson spike trains with rate $\tilde{r}(t)$ and the average value was taken.

Expected van Rossum distances between the inhomogeneous Poisson neurons and the target spike trains were calculated. This can be accomplished by first filtering the Poisson neuron firing rate with the exponential filter of the van Rossum distance. The resulting function is equivalent to the expected function obtained by filtering and averaging very large ensemble of Poisson spike train realizations of the rate. Thus, the expected Van Rossum distance between a Poisson process with rate $\tilde{r}(t)$ and a spike train **u** is equal to

(36) $$\langle d(u,\tilde{r}(t))\rangle =\sqrt{\int_{0}^{T}{\left[u*h(t)-\tilde{r}(t)*h(t)\right]}^{2}dt\;}$$

## 2. Results

An evolutionary algorithm for fitting spiking neuron models to time varying signals has been presented. The system used a van Rossum metric between spike trains as a fitness function. Initially, the algorithm was applied to synthetic data to study its convergence properties. The algorithm was then applied to modeling *in-vitro* data with several adaptive two variable neuron models. The algorithm was then further tested on the optimization of a cascade neuron model consisting of a receptive field and aEIF model applied to auditory spike train data from zebra finch responding to conspecific song.

**Figure 5:**
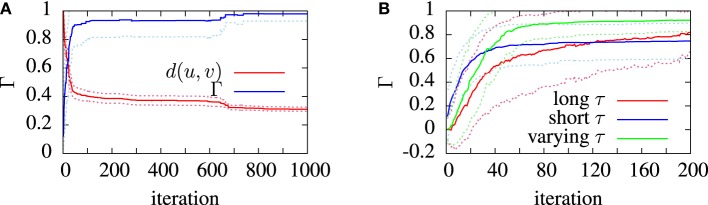
**Convergence of the van Rossum distance and coincidence factor in model finding runs.** These simulations used synthetic model data as targets. The convergence was measured and averaged over 20 identical runs. The algorithm used the van Rossum distance, *d*, between the experimental and model spike trains, *u* and $\tilde{u}$, as the fitness function. The van Rossum distance is normalized by its initial value. **(A)** shows the average performance of the algorithm. **(B)** shows the convergence behavior of the coincidence factor with different timescale choice in three cases; using a long timescale (*T*/2), a short timescale (1/*f*) and a varying timescale, decreasing from an initial value of *T*/2 down to 1/*f*. The dotted lines show the standard deviation from the mean (solid) lines.

The purpose of testing the algorithm on artificial target data was to show that the algorithm was capable of finding, near enough, a set of parameters which are known to exist somewhere in the search space. Figure 5 shows the convergence behavior of the algorithm in fitting an aEIF model to a data set consisting of 2 s of simulated aEIF response to a random current input. The population best van Rossum distance and the coincidence factor for the corresponding members were recorded at each iteration and averaged over 20 runs for this plot. As can be seen, a near perfect fit is achieved with a high degree of reliability. The coincidence factor consistently rose to a value close to one between the model and target on repeated runs of the algorithm on the target data generated by an aEIF model. A stringent coincidence window width of δ = 0.5 ms was used for this relatively easy optimization problem; the quantitative single neuron modeling competition used a value of δ = 2 ms in its benchmarking of fits to real *in-vitro* data.

The target parameters and the values obtained by the search algorithm are given in Table 2 together with the search range used by the algorithm to initialize the population. It is apparent that some variance exists in the parameters obtained although a high degree of accuracy was achieved with the target parameters. This could be due to the coincidence window of 0.5 being still relatively large or due to models such as the aEIF being rescalable to a dimensionless forms with fewer parameters (Touboul and Brette, 2008).

The effect of varying the van Rossum distance timescale on model convergence was investigated during the runs on artificial target data. In Figure 5B we see the evolution of the population best coincidence factor for different values of τ in the van Rossum distance. The three curves show the average convergence rate of the algorithm in three cases—using a constant long timescale, a constant short timescale and a varying timescale.

Using a varying timescale consistently improved convergence behavior over using a constant timescale. A short τ resulted in a rapid initial climb which leveled off relatively early. A long τ resulted in a slower rise in coincidence factor. The varying time scale allows the correct firing rate to be quickly found in the early stages of the search by using a wide timescale, leading to sharp improvements early on. Later, with a narrower timescale, the van Rossum distance switches to functioning more like a coincidence detector and fine tunes the model. The effect of a varying timescale on the resulting spike train functions is illustrated in Figure 2.

The performance results of the model fitting studies on the *in-vitro* data are summarized in Table 3. The best results for each model are similar those obtained in the study by Rossant et al. (2010) who used the same data. However, the exponential integrate-and-fire neurons excelled in our experiments. Without an adaptive threshold, the aEIF model is seen to perform considerably better than the integrate-and-fire models with either an adaptation current or an adaptive threshold, as can be seen in Table 3. This is likely due to the advantages offered by the exponential term in allowing fast spike initiation.

**Table 3:** **Performance and computational cost**.

	**〈Γ〉/Γ_*i*_**	**N^*o*^ Parameters**	**Computational cost**
aIF	0.63 ± 0.04	4	1.7
atIF	0.64 ± 0.06	5	1.8
aEIF	0.74 ± 0.06	9	8.3
a^2^EIF	0.78 ± 0.03	11	8.4
aEIF approximation	0.74 ± 0.06	9	5.6
a^2^EIF approximation	0.77 ± 0.03	11	5.7
MAT^*^	0.59 ± 0.02	5	1.1
Izhikevich	0.38 ± 0.03	5	2.1

The table shows the mean of the best coincidence factors recorded on each trial for several different models on the Challenge A data set of the INCF quantitative single neuron modeling competition. The figures are averages ± a standard deviation across each of 13 trials and 10 parameter finding runs on each trial. The models were implemented with 4th order Runge-Kutta numerical integration schemes. The relative cost figures indicate the time taken to simulate that particular neuron model, with the required spike count, relative to a simple integrate-and-fire neuron. The ‘approximation’ values for the aEIF and a^2^EIF models were obtained using an approximation of the exponential term as described in Schraudolph (1998).

* means that we used the implementation of the model described in Kobayashi et al. (2009) rather than our own code.

**Figure 6:**
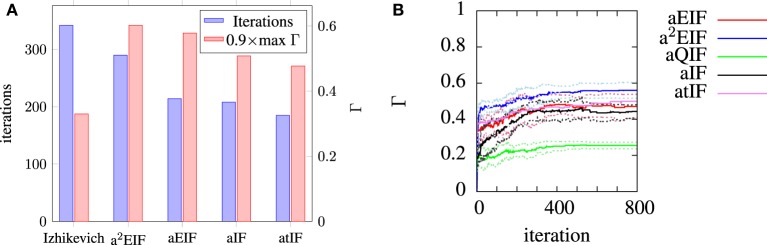
**(A)** Shows the average number of iterations to reach and not again fall below 90% of the maximum coincidence factor achieved on that run plotted with the maximum achieved coincidence factors for each neuron. We chose to plot the data in this way because the coincidence factor does not increase monotonically with decreasing van Rossum distance. **(B)** Shows the evolution of average population best coincidence factor averaged across trials for each model used for modeling the *in-vitro* data set. Note that the coincidence factor does not strictly increase because it is not the fitness function, but rather, the van Rossum distance is. The dotted lines indicate the standard deviation from the mean (solid) lines.

The a^2^EIF gave the highest predictive accuracy among the models studied. The computational cost of the model is only marginally higher than the aEIF model as the extra equation is linear. The aEIF model does however converge to 90% of the maximum achieved in considerably less iterations of the genetic algorithm than the a^2^EIF model. This is illustrated in Figure 6A, which shows the average number of iterations required on each model to reach 90% of the maximum achieved coincidence factor for, and not again drop below it. For this reason we chose to use the aEIF model in the *in-vivo* experiments, the results of which are presented in the next section. This figure was plotted because the coincidence factor does not consistently increase with decreasing van Rossum distance. Instead, it fluctuates up and down as changes which cause slight improvements in van Rossum distance sometimes cause a drop in coincidence factor.

In Table 3 the computational cost figures are scaled relative to a simple integrate-and-fire neuron responding to the same input stimulus. The exponential term in the two exponential IF neurons is the cause of their relatively high computational costs. Series approximations of exponentials, as performed by standard math libraries, require many floating point operations. A lower order approximation of the exponential carefully designed for the likely range of the exponent, as presented by Schraudolph (1998), can speed these models up dramatically with negligible changes in accuracy.

This approximation was tested. The performance and computational cost figures obtained with this method are shown in the table in brackets next to the figures for the aEIF and a^2^EIF neurons obtained using calls to the standard exponential function in the C math library. A speed increase of more than 30% is achieved with negligible change in predictive accuracy according to the coincidence factor calculated at a resolution of 2 ms. However, the computational cost is still more than five times that of a leaky integrate-and-fire neuron.

The MAT model was less computationally costly to simulate than the other models we studied as it is amenable to an analytical solution. We used the code published by Kobayashi et al. (2009) to simulate the MAT model results and this code calculates the analytic solution of the integrate and fire neuron. For the other spiking models, we used a 4th order Runge-Kutta numerical integration scheme.

### 2.1. *In-Vivo* neuron modeling results

**Figure 7:**
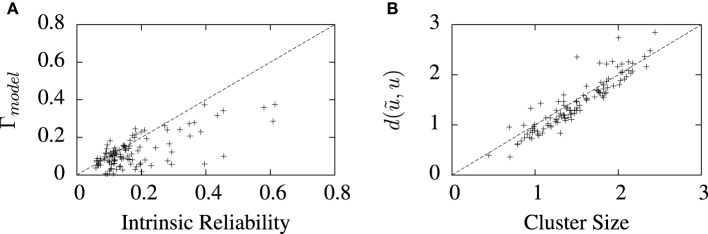
**Performance of the full model on 110 cell data sets plotted against measures of the reliability of the data under two metrics. (A)** Shows the best coincidence factor plotted against the intrinsic reliability for each cell. **(B)** Shows the best van Rossum distance plotted against the cluster size—the average inter-trial van Rossum distance of the experimental data set. ^+^refer to the individual data points; each ^+^corresponds to an individual cell in the cohort of cells studied.

The values of best coincidence factor and the best van Rossum distance obtained from the STRF-aEIF model on the validation data set are plotted in Figure 7 against corresponding estimates of the reliability of the data for each cell, that is, the average inter-trial coincidence factor and inter-trial van Rossum distance of the data. In Figure 7A the coincidence factor is plotted against the intrinsic reliability for each cell while in Figure 7B the van Rossum distance is plotted against the “cluster size”; this is the average inter-trial van Rossum distance of the set of experimental validation spike trains.

For many of the cells, the algorithm found solutions close to these theoretical values of the reliability of the data, particularly so for van Rossum distances. These limits are not hard upper limits however and models can exceed them in certain circumstances. For example, if a model spike train is found which lies somewhere in the center of a space whose edges are defined by the set experimental spike trains, then that spike train can have a lower average distance from all the experimental trains than the average inter-trial distance between the experimental trains. Such a scenario is likely to occur when the experimental recordings are particularly noisy and variable.

**Figure 8:**
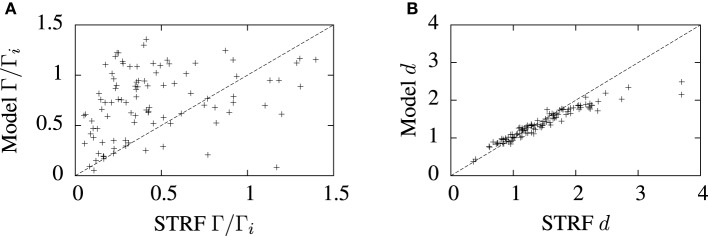
**Comparison of model predictions and normalized reverse correlation predictions on the validation set under three metrics; (A) the coincidence factor and (B) the van Rossum distance.** The average values of the coincidence factor scaled by the intrinsic reliability were Γ = 0.76 ± 0.08 for the STRF-aEIF models and Γ = 0.61 ± 0.05 for the STRF-poisson model. The STRF-aEIF model had an average better coincidence factor in 70.6% of cells. The average van Rossum distances were 1.39 ± 0.05 for the STRF-aEIF model and 1.50 ± 0.03 for the STRF-Poisson model with the STRF-aEIF performing better in 59% of cases. ^+^refer to the individual data points; each ^+^corresponds to an individual cell in the cohort of cells studied.

The predictive accuracy of the cascade STRF-aEIF model vs. normalized reverse correlation STRFs realized as spiking neurons using an inhomogeneous Poisson process is illustrated in Figure 8. While there is considerable variation, in general it can be seen from Figure 8A that the STRF-aEIF model on average achieved a better coincidence factor with the validation data than a STRF-Poisson cascade spiking model; it performed better in 70.6% of the data sets with an average performance factor of Γ = 0.76 ± 0.08 vs. Γ = 0.61 ± 0.05.

**Figure 9:**
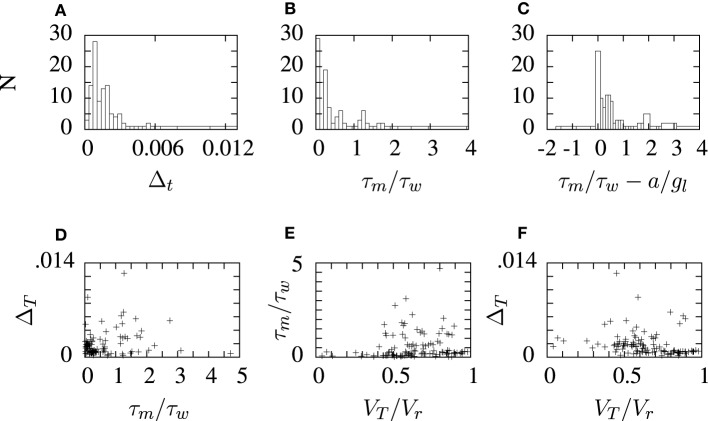
**Histograms and scatter plots of aEIF parameters obtained from the STRF-aEIF model optimization runs.** The ratios on the *x*-axis in panels **(B,C)** were chosen as they were presented as criterion for classifying the firing type of aEIF neurons in Naud et al. (2008). The scatter plots show the ratio of threshold to reset *V*_*T*_/*Vr*, the ratio of time constants τ_*m*_/τ_*w*_ and **(A)** shows the slope factor of the exponential term, Δ_*T*_, in the aEIF model. **(D–F)** show sample scatter plots of some of the parameters plotted against each other which were plotted to see if there may be any correlations present.

The aEIF neuron model parameters obtained by fitting the STRF-aEIF model are collected and the distribution of some of these parameters are plotted in histograms in Figure 9. Several of the parameters are also shown plotted against eachother in scatter plots although no clear relationships were found and parameters tended to take values centered around the initial regions used by the algorithm. The slope factor of the exponential term, Δ_*t*_, determines how big of an impact the exponential term has on the dynamics of the model. Many of the estimated values of Δ_*t*_ are quite close to zero and less than the sample value quoted by Brette and Gerstner (2005) which suggests that the exponential term of the aEIF models found here typically did not have as strong of an effect in the model dynamics as the model used in Brette and Gerstner (2005).

The runtime of the algorithm was on the *in-vivo* data was very variable and dependent on the length of the training data set, the spiking frequency, and on the choice of population size and maximum iterations in the genetic algorithm. However, it was generally a costly process. Typically, running the algorithm for 1000 iterations with a population size of 240 took approximately half an hour on average using a desktop PC with a 3.4 GHz AMD athlon CPU and a tesla C1060 GPU. The GPU was used to simulate and compute the fitness of each population member in parallel on each iteration. A more universal version of the code which did not require the GPU but instead used OpenMP directives to parallelize the code on the CPU, achieved results which were several times slower. It took roughly 3 h to run the same simulation on the CPU only.

## 3. Discussion

An optimization tool for generating models of spiking neurons from both *in-vitro* and *in-vivo* electrophysiological data has been presented. The optimization algorithm used is a hybrid genetic algorithm, a heuristic optimization algorithm capable of globally optimizing the parameters of a function even when no gradient information is available. This is particularly useful with the nonlinear spiking neuron models whose behaviors depend in a complicated way on their parameters.

The van Rossum distance was used as a fitness function. A continuous objective function is useful in a continuous parameter space since incremental steps toward convergence lead to incremental improvements in the value of the function. We found that the choice of timescale in the van Rossum distance made it an adaptable fitness function. A large timescale quickly narrows the search to models with the correct firing rate while a shorter timescale allows fine tuning of the model parameters.

Several other authors have studied optimization methods for spiking neurons. Gerken et al. (2006) also used a genetic algorithm. However, their work was restricted to a case where the input current was known and the algorithm used the mean squared error between the real and model *I* − *f* curves as a fitness function rather than a spike train metric. Rossant et al. (2010) applied an evolutionary algorithm, the particle swarm method, to the same *in-vitro* spike train data as as is considered here and uses the coincidence factor between spike trains as a fitness function; however, they did not explore the *in-vivo* case.

Our results on the *in-vitro* data are roughly in agreement with Rossant et al. (2010). We also saw that neuron models featuring both adaptive and non-linear model components achieved a better fit of the data than the other spiking models. The a^2^EIF model presented here yielded the greatest predictive accuracy but was the most costly to simulate and second most costly to fit. As noted elsewhere (Jolivet et al., 2008b; Kobayashi et al., 2009; Rossant et al., 2010), adaptation is a crucial ingredient in an accurate model.

When we applied our algorithm to fit a STRF-neuron cascade model to *in-vivo* extracellular data, the model was optimized by evolving both the receptive field and the neuron parameters in tandem. The resulting models had, on average, a greater predictive accuracy than receptive field estimates of the firing rate in terms of the expected coincidence factor and van Rossum distances of the rate models from the target data.

The optimized aEIF neuron model parameters do not vary very much across all the cells studied: Figure 9 shows histograms of some of the aEIF parameters found for each cell. It would be interesting as future work to study the effect of optimizing the STRFs with a constant set of typical neuronal parameters used across all cells. In any case, the main benefit of a cascade model is that introduces spike rate adaptation effects which are absent in a linear rate response model.

**Figure 10:**
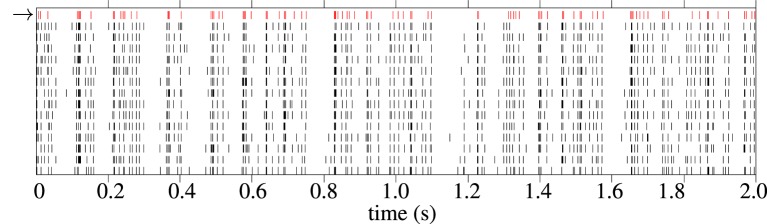
**Raster plot showing 2 s of spike trains in response to Zebra Finch conspecific song.** The first spike train, in red with the arrow next to it, is a spike train generated by the cascade model. The 12 black spike trains are individual trials from experimental recordings The cell is “pupi01414_10,” taken from the CRCNS aa-2 data set (Theunissen et al., 2011).

An example raster plot showing a model response and corresponding experimental spike train responses from a typical cell from the *in-vivo* data is shown in Figure 10. The spike trains generated by the model generally have fewer spikes than the average spike count of the experimental trials. This is because the fitness function tends to favor spike trains with fewer but more accurate spikes during periods of high variability in the target data; a model cannot match the spike rate without increasing the error. One solution to this would be to fit to a single target spike train, such as the central spike train described in Julienne and Houghton (2013).

A drawback of our method for fitting the *in-vivo* model is that estimating STRFs using a genetic algorithm is computationally costly: STRFs typically have several 100 parameters and a large population of several 100 STRFs must be used in a genetic algorithm. Nonetheless, the biological justification for the STRF models relies on an interpretation of the STRF parameters as a proxy for synapse strengths in the local neuronal circuit and the model presented here demonstrates that that interpretation can be usefully extended to an actual neuronal model.

### Conflict of interest statement

The authors declare that the research was conducted in the absence of any commercial or financial relationships that could be construed as a potential conflict of interest.
