# Supplementary material for: Parameter estimation of neuron models using in-vitro and in-vivo electrophysiological data
Source: Front Neuroinform. 2015 Apr 20;9:10. doi: 10.3389/fninf.2015.00010 (PMC4403314; doi:10.3389/fninf.2015.00010)
Supplement: Supplementary file 1 [file Table1.PDF]

Supplementary Material: Parameter estimation of neuron models using *in-vitro* and *in-vivo* electrophysiological data

Eoin Lynch [1,2,*], **Conor Houghton** [2]

[1] *School of Mathematics, Trinity College Dublin, Dublin, Ireland*
[2] *Department of Computer Science, University of Bristol, United Kingdom*

Correspondence*:
Eoin Lynch
School of Mathematics, Trinity College Dublin, Dublin 2, Ireland,
eplynch@maths.tcd.ie

1 SUPPLEMENTARY TABLES AND FIGURES

Supplementary Table 1. Parameters of each model and initial search regions used by the genetic algorithm to generate initial populations in both the *in-vitro* and *in-vivo* model fitting experiments. The initial parameter values of the population are drawn from uniform distributions on the intervals indicated.

Model	parameter	unit	initial search regions
aEIF	τ_m	ms	$\in [5, 20]$
	E_L	mV	$\in [-60, -80]$
	Δ_T	mV	$\in [0, 2]$
	V_T	mV	$\in [-60, -40]$
	b	nS	$\in [-5, 5]$
	α	nA	$\in [0.0, 0.1]$
	τ_w	ms	$\in [10, 150]$
	V_r	mV	$\in [-60, -90]$
	V_c	mV	fixed 0 mV
	R	Ω	$[1 \times 10^7, 1 \times 10^8]$
a^{2}EIF	above parameters plus:		
	τ_t	ms	$\in [20, 120]$
	β	mV	$\in [0, 0.3]$
aIF	τ_m	ms	$\in [5, 20]$
	τ_w	ms	$\in [10, 150]$
	α	mV	$\in [0, 20]$
	V_c	mV	fixed -50 mV
	$V_r (= E_L)$	mV	fixed -70 mV
	R	Ω	$[1 \times 10^7, 1 \times 10^8]$
$7.27 \times 10^7 \Omega$			
atIF	tau_m	ms	$\in [5, 20]$
	τ_t	ms	$\in [10, 150]$
	α	mV	$\in [0, 10]$
	b	-	$\in [0, 1]$
	V_{c0}	mV	fixed -50 mV
	$V_r (= E_L)$	mV	fixed -70 mV
	R	Ω	$\in [1 \times 10^7, 1 \times 10^8]$
aQIF	a	-	$\in [0, 0.2]$
	b	-	$\in [0, 2]$
	c	mV	$\in [-75, -65]$
	d	-	$\in [0, 500]$
STRF	$h(\tau, \omega)$		NRC solution parameters ± a random purturbation of each parameter by between 0 and 50% of its amplitude.
